# Telomere Length but Not Mitochondrial DNA Copy Number Is Altered in Both Young and Old COPD

**DOI:** 10.3389/fmed.2021.761767

**Published:** 2021-11-24

**Authors:** Sandra Casas-Recasens, Nuria Mendoza, Alejandra López-Giraldo, Tamara Garcia, Borja G. Cosio, Sergi Pascual-Guardia, Ady Acosta-Castro, Alicia Borras-Santos, Joaquim Gea, Gloria Garrabou, Alvar Agusti, Rosa Faner

**Affiliations:** ^1^Centro de Investigación Biomédica en Red de Enfermedades Respiratorias (CIBERES), Madrid, Spain; ^2^Institut d'Investigacions Biomediques August Pi i Sunyer (IDIBAPS), Barcelona, Spain; ^3^Respiratory Institute, Hospital Clinic, Barcelona, Spain; ^4^Department of Pneumology, University Hospital Son Espases, Palma de Mallorca, Spain; ^5^Institut d'Investigació Sanitària Illes Balears (IdISBa), University Hospital Son Espases, Palma de Mallorca, Spain; ^6^Servei de Pneumologia, Hospital del Mar - IMIM, Barcelona, Spain; ^7^Universitat Pompeu Fabra, Barcelona, Spain; ^8^Pulmonary Service and Research Institute, Doce de Octubre University Hospital, Madrid, Spain; ^9^ISGlobal, Barcelona, Spain; ^10^Faculty of Medicine and Health Sciences, University of Barcelona, Barcelona, Spain; ^11^Muscle Research and Mitochondrial Function Laboratory, Internal Medicine Service, Hospital Clinic of Barcelona, Barcelona, Spain; ^12^CIBERER-Spanish Biomedical Research Centre in Rare Diseases, Madrid, Spain

**Keywords:** telomeres, mitochondrial DNA, chronic bronchitis, emphysema, ageing

## Abstract

Accelerated ageing is implicated in the pathogenesis of respiratory diseases as chronic obstructive pulmonary disease (COPD), but recent evidence indicates that the COPD can have roots early in life. Here we hypothesise that the accelerated ageing markers might have a role in the pathobiology of young COPD. The objective of this study was to compare two hallmarks of ageing, telomere length (TL), and mitochondrial DNA copy number (mtDNA-CN, as a surrogate marker of mitochondrial dysfunction) in young (≤ 50 years) and old (>50 years) smokers, with and without COPD. Both, TL and mtDNA-CN were measured in whole blood DNA by quantitative PCR [qPCR] in: (1) young ever smokers with (*n* = 81) or without (*n* = 166) COPD; and (2) old ever smokers with (*n* = 159) or without (*n* = 29) COPD. A multivariable linear regression was used to assess the association of TL and mtDNA-CN with lung function. We observed that in the entire study population, TL and mtDNA-CN decreased with age, and the former but not the latter related to FEV_1_/FVC (%), FEV_1_ (% ref.), and DLCO (% ref.). The short telomeres were found both in the young and old patients with severe COPD (FEV_1_ <50% ref.). In addition, we found that TL and mtDNA-CN were significantly correlated, but their relationship was positive in younger while negative in the older patients with COPD, suggesting a mitochondrial dysfunction. We conclude that TL, but not mtDNA-CN, is associated with the lung function impairment. Both young and old patients with severe COPD have evidence of accelerated ageing (shorter TL) but differ in the direction of the correlation between TL and mtDNA-CN in relation to age.

## Introduction

Chronic obstructive pulmonary disease (COPD) is a major public health problem because of its high prevalence (>10% of adults), rising incidence (third current global cause of death), and associated costs (*circa* 38 billion annually in the European Union [EU] only) (1). It has been traditionally understood as self-inflicted disease caused by tobacco smoking and characterised by an accelerated decline of lung function with age (2, 3), suggesting that smoking can accelerate the physiological lung ageing (4–6). Recent research, however, has clearly shown that lung development abnormalities, both before or after birth, limit the peak lung function value achieved in early adulthood and can also lead to COPD later in life (7). As a result, there is great interest in understanding the pathobiology of COPD in young patients (8–10). We hypothesise that ageing pathways are dysregulated in young patients with COPD, a research question, that to our knowledge, has not been investigated to date.

Telomere attrition and mitochondrial dysfunction are two well-recognised and inter-related molecular hallmarks of ageing (4) since, on the one hand, telomere attrition leads to mitochondrial biosynthesis reprogramming and, on the other, the latter induces telomere attrition (11). Further, their correlation can provide insights into the regulation of the ageing process (11). Previous studies have shown that both telomere length (TL) (5, 12–16) and mitochondrial DNA copy number (mtDNA-CN, as a surrogate marker of mitochondrial dysfunction) (17) in the circulating leukocytes are reduced in the old patients with COPD, but they have not been investigated in young patients with COPD so far. Likewise, their relationship with the smoking history of the patient, the severity of airflow limitation, or degree of emphysema present have not been studied in this setting either. Here, we investigated the relationship between TL and mtDNA-CN with several lung function indices and smoking exposure in peripheral blood of young (35–50 years.) and old (>50 years.) ever smokers, with or without COPD, as well as their correlation in this setting.

## Materials and Methods

### Study Population and Ethics

Following the operational definition of Early COPD by Martinez et al. (9), we defined young as those individuals between 35 and 50 years of age, and old as those over 50 years (18). All the individuals were current or former smokers, with preserved ratio between the forced expiratory volume in one second and the forced vital capacity FEV_1_/FVC (controls) or with COPD (cases, FEV_1_/FVC <0.7) (Table 1). All the individuals were recruited from the centers participating in the CIBERES COPD research program in Spain (19–21). All the participants signed their informed consent, and the Ethics Committee of Hospital Clinic (Barcelona, Spain) approved the study (HCB-2018/135). All experimental determinations were performed in our laboratory at IDIBAPS by the same operator.

**Table 1:** The characteristics of the participants [mean ± SD or number (%)].

	**Young ≤50 yrs**.			**Old >50 yrs**.			
	**Controls *n* = 166**	**COPD *n* = 81**	***p*-value**	**Controls *n* = 29**	**COPD *n* = 159**	***p*-value**	**p. overall**
Age, years	43.8 ± 4.41	46.5 ± 3.52	<0.001	61.0 ± 7.18	65.7 ± 7.69	0.002	<0.001
Males, *n* (%)	83 (50.0%)	55 (67.9%)	0.006	17 (58.6%)	133 (83.6%)	0.005	<0.001
Body Mass Index, Kg/m^2^	26.7 ± 4.82	28.3 ± 6.34	0.064	27.2 ± 4.33	27.3 ± 5.33	0.96	0.16
Smoking status			0.163			<0.001	<0.001
Current smokers, *n* (%)	128 (77.1%)	55 (67.9%)		23 (79.3%)	63 (39.6%)		
Former smokers, *n* (%)	38 (22.9%)	26 (32.9%)		6 (20.7%)	96 (60.4%)		
Smoking exposure, pack-years	25.2 ± 13.9	31.3 ± 15.6	0.005	32.8 ± 19.1	54.4 ± 24.4	<0.001	
FEV_1_, % ref	98.0 ± 13.9	70.8 ± 21.5	<0.001	94.1 ± 12.0	52.2 ± 20.8	<0.001	<0.001
FVC, % ref	99.7 ± 14.1	92.5 ± 21.4	0.007	84.0 ± 12.4	77.3 ± 21.3	0.037	<0.001
FEV_1_/FVC, %	79.5 ± 5.46	58.5 ± 10.5	<0.001	75.4 ± 3.71	50.6 ± 12.5	<0.001	<0.001
DLCO, % ref.	91.6 ± 13.7	82.7 ± 23.5	0.003	98.0 ± 18.6	50.4 ± 27.9	<0.001	<0.001
GOLD grades			<0.001			<0.001	<0.001
grade 1–2, *n* (%)	–	66 (81.5%)		–	77 (48.4%)		
grade 3–4, *n* (%)	–	15 (18.5%)		–	82 (51.6%)		
log(TL)	3.71 ± 0.19	3.64 ± 0.20	0.012	3.57 ± 0.25	3.43 ± 0.22	0.01	<0.001
log(mtDNA-CN)	3.54 ± 0.54	3.53 ± 0.63	0.906	3.18 ± 0.30	3.16 ± 0.37	0.74	<0.001

### Characterisation of Participants

The demographics and symptoms were registered using standardised questionnaires. Patients with α1-antitrypsin deficiency were excluded. Spirometry and the single-breath carbon monoxide diffusing capacity of the lung (DLCO) were measured following the international recommendations (22, 23). The reference values were those of the Global Lung function Initiative (GLI) (24). A DLCO is a well-established marker of emphysema (25, 26) so, as previously described, the presence of emphysema was defined as DLCO lower than 60% ref., and the absence of emphysema as a DLCO higher than 80% ref. (27, 28).

### DNA Extraction

Blood was collected using ethylenediamine tetraacetic acid (EDTA) anticoagulation tubes and stored at −80°C until analysis. DNA was extracted using the QiAmp Blood DNA kit according to the supplier's instructions (Qiagen, Germany). DNA purity and concentration were assessed with a Nanodrop (Life Technologies, CA, USA) and a Qubit 4 Fluorometer (Life Technologies, CA, USA), respectively.

### TL Measurement

Telomere length was measured in DNA by quantitative PCR (qPCR) following the method described by Cawthon (29). Briefly, the number of telomere repeats and that of albumin (a single copy gene) were assessed in the same qPCR tube (as shown in the Supplementary Material). TL is expressed as a relative telomere to single copy gene ratio as described by Cawthon (29).

### mtDNA-CN Quantification

To determine mtDNA-CN in DNA, the mitochondrial 12S ribosomal RNA (mt12SrRNA) gene and the nuclear-encoded *RNAse P* gene were simultaneously determined in the same qPCR tube, as previously described (30) (as shown in the Supplementary Material). The mtDNA-CN was calculated as the ratio 12SrRNA mtDNA copies/RNAseP DNA copies.

### Statistical Analysis

The results are presented as number, percentage, mean ± SD, or median (95% *CI*). The groups were compared using the Kruskal–Wallis, Chi-square, or Fisher's exact tests, as appropriate. A multivariable regression analysis was used to explore the association of three lung function variables (FEV_1_ % ref., FEV_1_/FVC (%), and DLCO % ref.) with the two hallmarks of ageing investigated here (TL and mtDNA-CN, both previously log transformed to approximate a normal distribution). Initially, all the models were adjusted by age, sex, smoking status (current/former), and cumulative smoking exposure (pack-year); then, the contribution of each of these four covariates to the model was examined by comparing the goodness of fit of the different methods. The final models presented here included only those covariates that improved the fitting of the data significantly. The effect size was inferred from the estimate of the linear regression (β coefficient) and its 95% *CI*, that informs on the change in the outcome variable (TL or mtDNA-CN) for a unit change in the explanatory variable, holding all the other variables in the model constant. All the models with the estimates and *CI*s are provided in the Supplementary Tables 1–3. A *p*-value lower than 0.05 was considered statistically significant. All the calculations were performed using R with custom scripts, with figures being produced using its ggplot2 (31) and forestmodel (32) packages.

## Results

### Characteristics of Participants

In this analysis, we included 240 patients with COPD (81 young and 159 old) and 195 controls (166 young and 29 old) (Table 1). The patients with COPD were slightly older than corresponding controls and included a higher proportion of men. Airflow limitation was mild in younger and moderate-severe in the older patients with COPD, and DLCO % ref. was mildly reduced in the younger but severely impaired in the older patients with COPD. As previously described (10), our population of young ever smoker controls included some individuals with abnormal FEV_1_ % ref. and DLCO % ref. values despite a preserved FEV_1_/FVC ratio, who may currently qualify as pre-COPD subjects (8).

### TL and mtDNA-CN as Ageing Markers in the Entire Study Population

**Figure 1:**
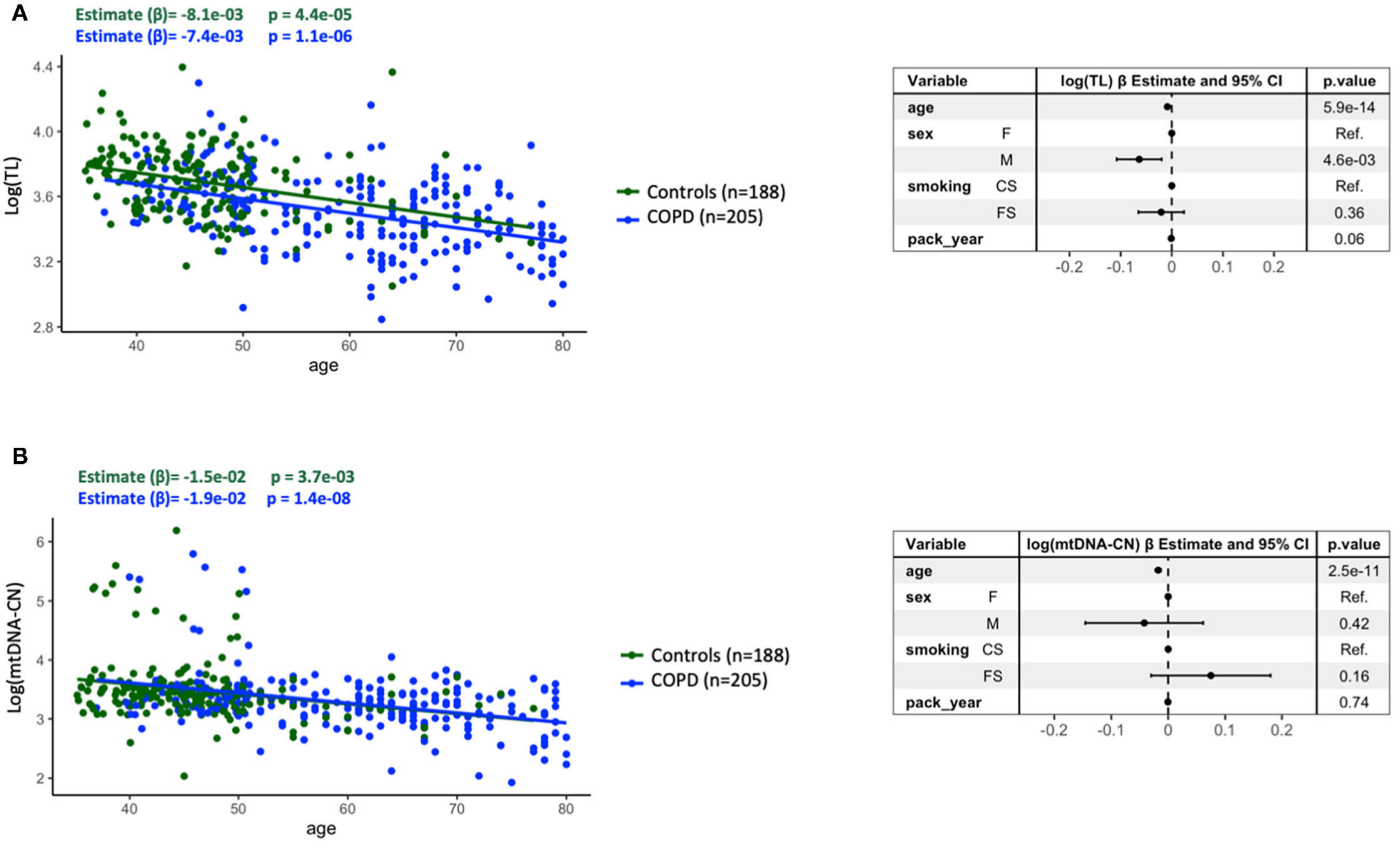
Relationship between telomere length [log(TL)] **(A)** or mitochondrial DNA copy number [log(mtDNA-CN)] **(B)**, both log transformed, and age in the entire study population, stratified by controls (*n* = 188) (green dots and line) and patients with COPD (*n* = 205) (blue dots and line). Forest plots of the linear regression models of all individuals (*n* = 393) presenting the point estimates and 95% *CI* (whiskers) of the change in log(TL) or log(mtDNA-CN) when adjusted for the potential confounders. For further explanations, see text.

Figure 1 shows that both TL and mtDNA-CN were negatively correlated with age (*p*-values = 5.9E-14 and 2.5E-11, respectively), confirming that both were indeed hallmarks of ageing in the population studied here. These associations were present in both controls and patients with COPD (*p*-values = 4.4E-05 and 1.1E-06 for TL and *p*-values = 3.7E-03 and 1.4E-08 for mtDNA-CN) (Figure 1). TL attrition with age was more pronounced in men, and cumulative smoking exposure (pack-year) just failed to reach statistical significance (*p* = 0.06) (Figure 1A). By contrast, neither sex, smoking status (current/former), or cumulative smoking exposure (pack-year) significantly influenced the relationship of mtDNA-CN with age (Figure 1B).

### Relation Between the Ageing Hallmarks and Lung Function in the Entire Study Population

**Figure 2:**
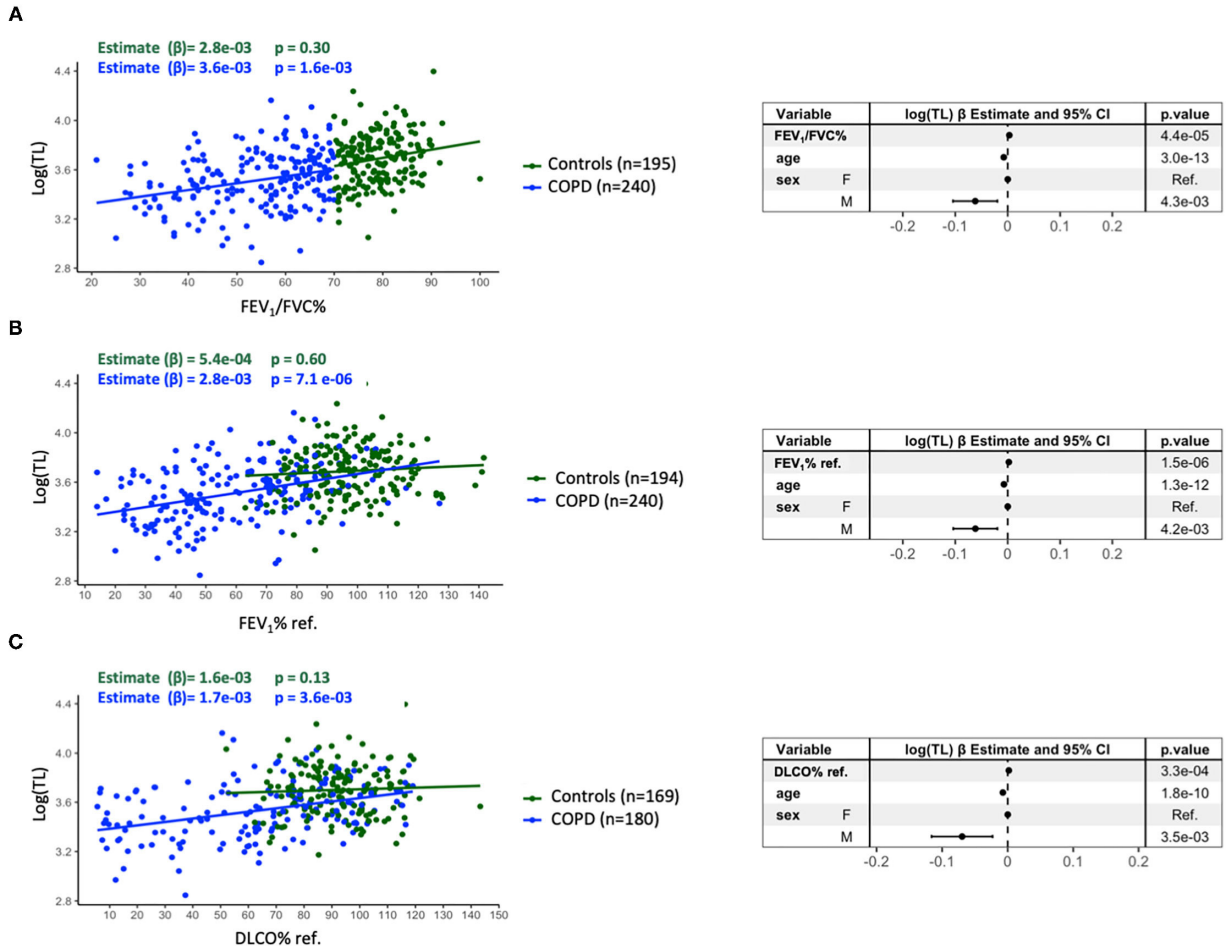
Relationship between the TL [log(TL)] and FEV_1_/FVC% **(A)**, FEV_1_% ref. **(B)**, and DLCO% ref. **(C)**. The blue dots identify the patients with COPD (**A**: *n* = 240, **B**: *n* = 240, **C**: *n* = 180) whereas the green dots correspond to controls (**A**: *n* = 195, **B**: *n* = 194, **C**: *n* = 169). Forest plots of the linear regression models of all individuals (**A**: *n* = 435, **B**: *n* = 434, **C**: *n* = 349) presenting the point estimates and 95% *CI* (whiskers) of the change in log(TL) when adjusted for the potential confounders. For further explanations, see text.

Figure 2 shows that, in the multivariable analysis of the entire study population, shorter telomeres were associated with lower FEV_1_/FVC ratio [*estimate* (β) = 3E-03 (95% *CI*: 1.6E-03, 4.5E-03), *p* = 4.4E-04], lower FEV_1_ % ref. [β = 2.1E-03 (95% *CI* 1.2E-03, 2.9E-03), *p* = 1.5E-06], and lower DLCO % ref. [β = 1.6E-03 (95% *CI*: 7.4E-04, 2.5E-03), *p* = 3.3E-04] values, with age and sex being significant covariates in the model. Of note, these relationships remained in the patients with COPD (Figure 1, blue dots) but did not in controls (green dots). If we use the lower limit of normal, instead of a fixed FEV_1_/FVC ratio <0.7, we observed similar results (Supplementary Figure 1). On the other hand, mtDNA-CN was not related to any of these three lung function indices (Supplementary Figure 2).

### Comparison of Young vs. Old Patients With COPD

**Figure 3:**
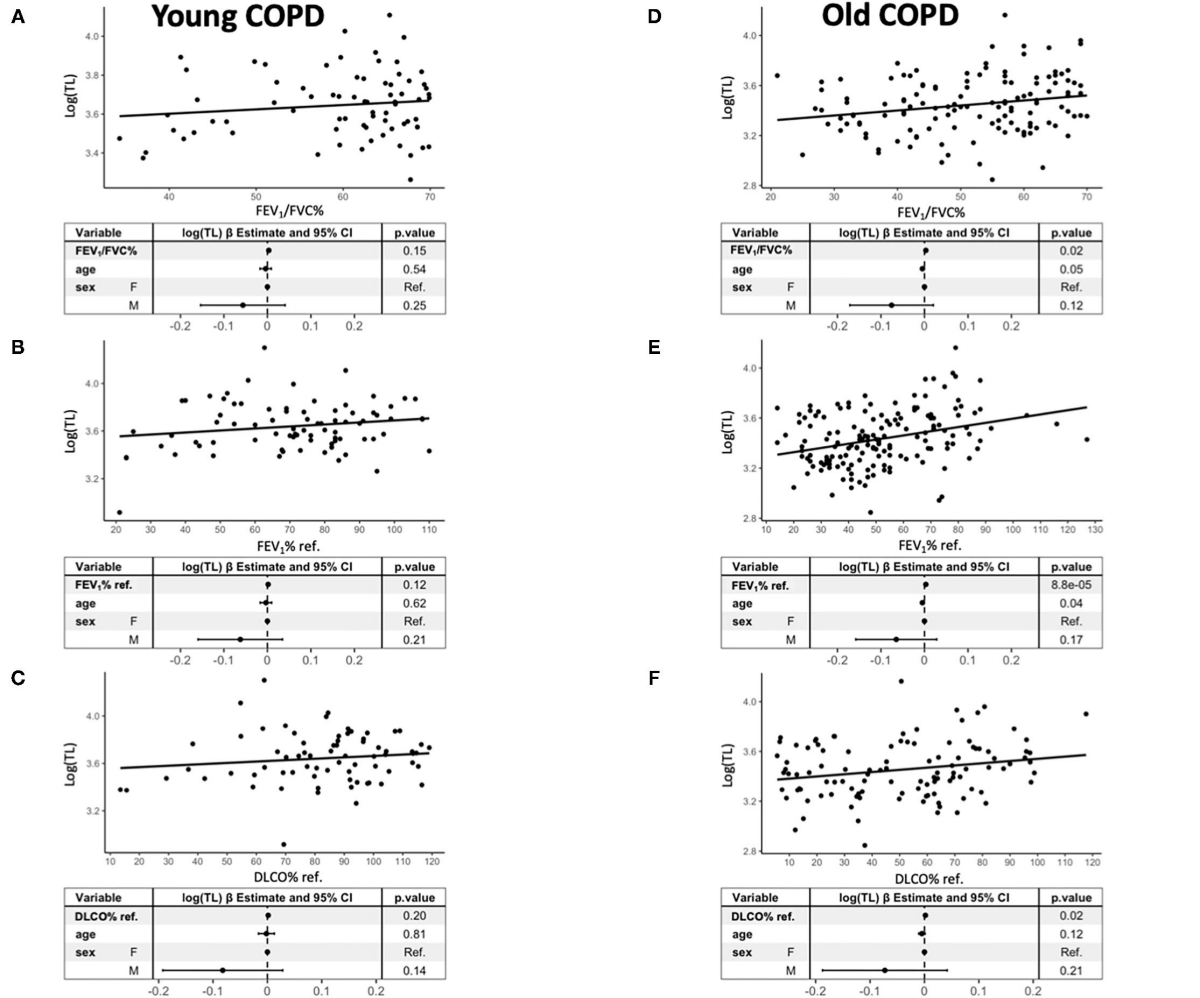
Relationship between TL [log(TL)] and FEV_1_/FVC% **(A,D)**, FEV_1_% ref. **(B,E)**, and DLCO% ref. **(C,F)** in young (left) (**A**: *n* = 81, **B**: *n* = 81, **C**: *n* = 72) and old (right) patients with COPD (**A**: *n* = 159, **B**: *n* = 159, **C**: *n* = 108). Forest plots of the linear regression models presenting the point estimates and 95% *CI* (whiskers) of the change in log(TL) when adjusted for the potential confounders. For further explanations, see text.

Figure 3 presents the relationship between TL and these three lung function variables in young (left) and old (right) patients with COPD. As in the entire population or patients with COPD at large, TL was significantly related to FEV_1_/FVC ratio [β = 3.2E-03 (95% *CI* 5.5E-04, 5.9E-03), *p* = 0.02], FEV_1_ % ref. [β = 3.2E-03 (95% *CI* 1.6E-03, 4.8E-03), *p* = 8.8E-05], and DLCO % ref. [β = 1.8E-03 (95% *CI* 2.6E-04, 3.3E-03), *p* = 0.02] in old patients (Figure 3, right panels), but failed to reach the statistical significance in younger patients [FEV_1_/FVC ration [β = 3.2E-01 (95% *CI* −0.11, 7.5E-03), *p* = 0.15], FEV_1_ % ref. [β = 0.17 (95% *CI* −4.4E-04, 3.8E-03), *p* = 0.12], and DLCO % ref. [β = 0.14 (95% *CI* −8.1E-04, 3.7E-03), *p* = 0.02)] (Figure 3, left panels). Supplementary Figure 3 shows that mtDNA-CN was not related to any lung function variable in old or young patients with COPD. These associations were not influenced by smoking.

**Figure 4:**
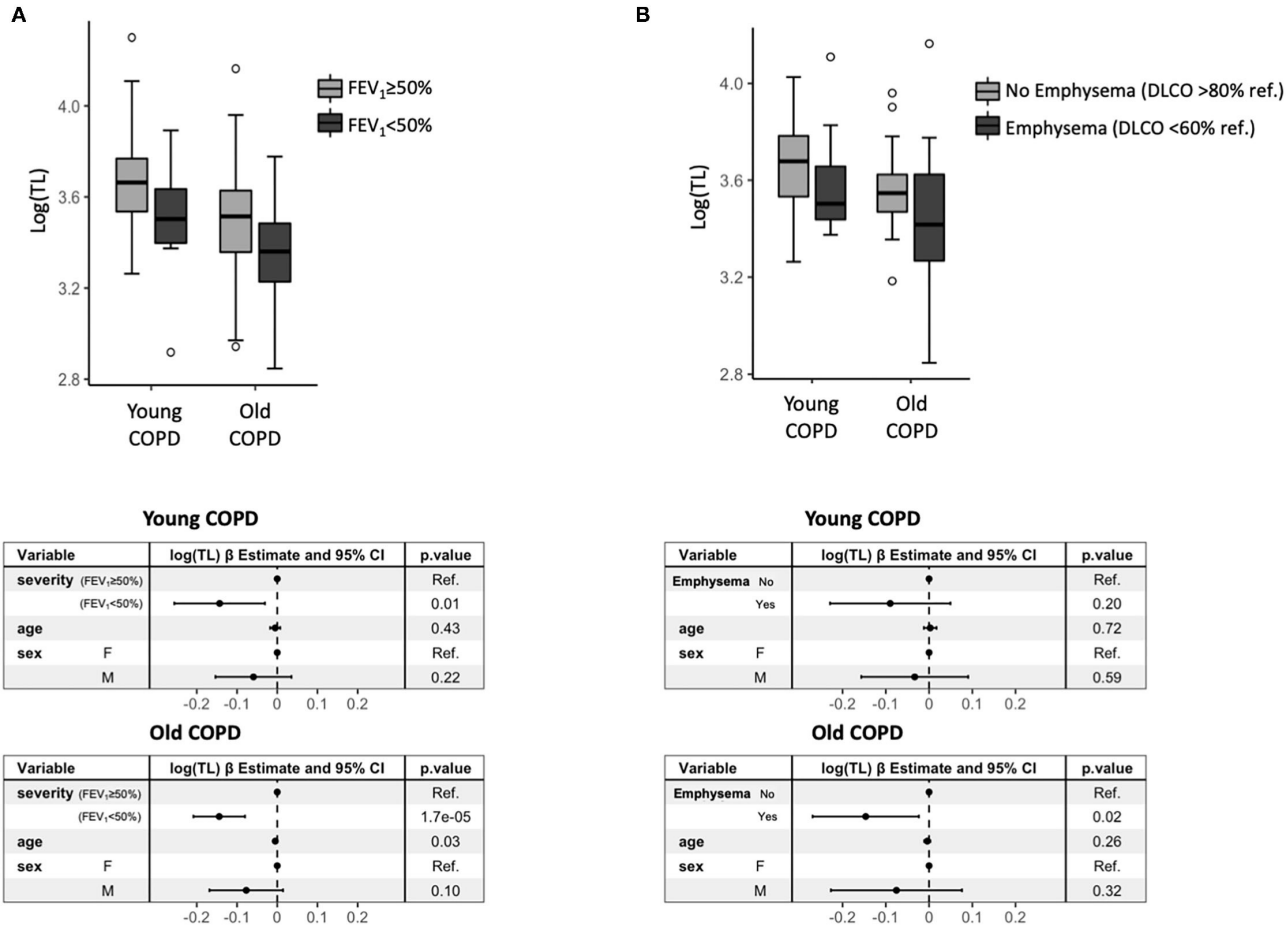
**(A)** A box plot of log(TL) by severity of airflow limitation in the patients with COPD stratified by young (*n* = 81) and old (*n* = 159), and corresponding forest plots (bottom) of the linear regression model presenting the point estimates and 95% *CI* (whiskers) of the change in log(TL) when adjusted for the potential confounders. **(B)** A box plot of log(TL) according to the presence (DLCO % ref. <60%) or absence (DLCO % ref. > 80%) of emphysema in the patients with COPD stratified by young (*n* = 55) and old (*n* = 78), and the corresponding forest plots (bottom) of the linear regression model presenting the point estimates and 95% *CI* (whiskers) of the change in log(TL) when adjusted for the potential confounders.

Figure 4A shows that TL was significantly shorter in patients with severe-very severe airflow limitation (FEV_1_ <50% ref.) than patients with mild-moderate airflow limitation (FEV_1_ ≥50% ref.), both in young and old patients with COPD. By contrast, mtDNA-CN was not different by airflow limitation severity in young or old patients (Supplementary Figure 4A). Figure 4B shows that the presence of emphysema in the patients with COPD, as indicated by a DLCO <60% ref., was associated with shorter telomeres in old patients with COPD but failed to reach the statistical significance in young patients (Figure 4B).

**Figure 5:**
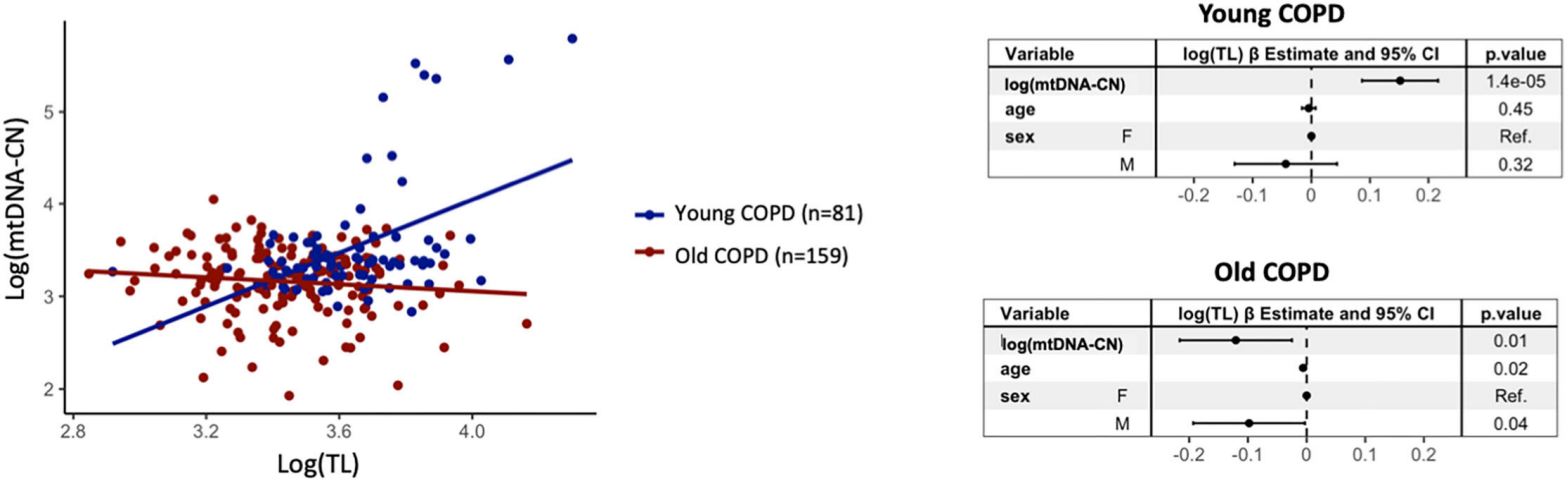
A scatter plot of log(TL) vs. log(mtDNA-CN) in young (*n* = 81) (dark blue dots) and old (*n* = 159) (red dots) patients with COPD, and the corresponding forest plots of the linear regression model estimates. For further explanations, see text.

Finally, Figure 5 shows that TL and mtDNA-CN in the patients with COPD were significantly related but the direction of such relationship was positive in young patients [β = 0.15, 95% *CI*: (8.6E-02, 0.22) *p* = 1.4E-05] and negative in older ones [β = −0.12, 95% *CI*: (−0.22, −2.5E-02), *p* = 0.01]. This different sign of correlation was preserved when the individuals with mtDNA-CN > 2 SDs of the mean, were removed from the analysis (Supplementary Figure 5).

## Discussion

Our study confirms previous reports that showed that TL and mtDNA-CN are hallmarks of ageing in the population since both decrease significantly with age (33). Yet, it extends previous observations by showing that TL (but not mtDNA-CN) relates to worse lung function in the population at large, in the patients with COPD (but not in ever smoking controls), as well as in the old patients (but not in young ones), and in patients with severe airflow limitation (both young and old). It also shows that the relationship between TL and mtDNA-CN was different in the young and old patients with COPD, suggesting different regulation of the telomere-mitochondria axis in these two age groups.

### Previous Studies

Several studies have previously investigated TL and mtDNA-CN in patients with COPD. Telomerase mutations and telomere attrition are described in alveolar type II and endothelial cells in patients with severe emphysema (34–36) and in mice models of smoking exposure (37). Other studies reported shortened telomeres (15) in old patients with COPD vs. controls (12, 13, 15, 16) and a modest association between TL and FEV_1_ % ref. (13), albeit the latter was not reproduced in other investigations (13–15, 38, 39). Recently, a longitudinal study in patients with COPD (40) showed an association between the accelerated telomere shortening, progressive worsening of the pulmonary gas exchange, and all-cause mortality risk. Moreover, some studies have included young and old individuals (14, 32) but none of them have stratified the analysis by age group, nor by the presence of COPD according to age.

However, compared with non-smokers, patients with COPD showed reduced mtDNA levels in exhaled breath, urine, and peripheral leucocytes (17, 41, 42). Finally, other approaches, as ageing clocks, are used to assess the relation between ageing and lung function in older ever/never smokers (43). None of these previous studies, however, investigated TL or mtDNA-CN in the young patients with COPD.

### Interpretation of Novel Findings

By studying a large population of young and old ever smokers, with and without COPD, our results provide a more comprehensive perspective on the relationship between these two well-established hallmarks of ageing (TL and mtDNA-CN) and lung function than the previous studies. We found that, as expected, both decreased with age but, in contrast, they exhibit a different relationship with lung function. While TL related to FEV_1_/FVC, FEV_1_ % ref., and DLCO % ref. in the population at large (patients and controls), in the patients with COPD (importantly, not in controls), old patients (not in young ones), and in patients with severe airflow limitation (both young and old), mtDNA-CN did not. This suggests the involvement of specific ageing mechanisms (telomere shortening) in the lung function abnormalities that characterise COPD. This may be particularly relevant in the young individuals since we observed here, for the first time to our knowledge, that telomere shortening also occur in the young patients with COPD with severe airflow limitation, a population that may be more susceptible to the environmental exposures associated to COPD and/or carry a genetic background predisposing them to telomere attrition. This finding agrees with the relationship between the accelerated telomere shortening and progressive worsening of pulmonary gas exchange previously described (40).

The observation of a correlation between the blood TL and airflow limitation suggests either that the circulating immune cells may have a pathobiologic role or that the effect of smoking exposure can be identified outside the lung (44, 45).

Although, as expected (46, 47), mtDNA-CN decreased significantly with age, it was not related to any lung function parameter. A previous study showed reduced mtDNA-CN levels in blood of COPD (17) but, at variance with our analysis, their results were not adjusted by age. In any case, our observations here illustrate that two different hallmarks of ageing (TL and mtDNA-CN) behave differently in relation to lung function, indicating again that not all the ageing mechanisms may be equally relevant for the pathobiology of COPD. This observation is in line with the results of Rutten et al. (38), who also measured different ageing markers in patients with COPD, albeit they only found TL to relate to lung function.

A somewhat surprising finding was that the multivariable analysis showed that cumulative smoking exposure in ever smokers did not affect the relationship between TL and lung function at any age. Yet, this observation is in keeping with the results of a meta-analysis of 18 longitudinal cohorts by Bateson *et al*. indicating that smoking does not accelerate leucocyte telomere attrition (48), and with those of Cordoba et al. showing that telomere shortening is associated with the airflow limitation independently of the smoking status (16). Importantly, it does not detract from the firmly established view that smoking is a key environmental risk factor for COPD (3); it only indicates that smoking *per se* does not influence the relationship between TL and lung function. In fact, the previous studies have shown a lack of correlation between packs year and FEV_1_ % ref. in the patients with COPD (49). Future studies should assess the prevalence of genetic variants associated to telomere length in the young and old patients with severe COPD (50).

Finally, it is known that TL and mtDNA-CN are inter-related. Whereas, mitochondrial dysfunction cause telomere attrition (51), telomere dysfunction activates the DNA damage response (p53) and leads to the repression of PGC-1α/PGC-1β and their downstream targets, that in turn drive a mitochondria biogenesis decline (52) and mitochondrial dysfunctions (11). Therefore, it has been suggested that investigating the correlations between TL and mtDNA-CN (as a surrogate marker of mitochondrial dysfunction) can provide insights into the balance between these two processes (53). Here we found that the relationship between TL and mtDNA-CN had a positive slope in the young patients with COPD, indicating that the young individuals with shorter telomeres have less mtDNA copies, but a negative one in the old patients with COPD, suggesting an accumulation of mitochondria with impaired respiratory chain because of impaired mitophagy (54), and/or compensatory mtDNA over-replication (55, 56). Interestingly, a similar loss of TL and mtDNA-CN co-regulation has been described in precancerous lesions (57, 58). It is well-established that lung cancer is more prevalent in (old) patients with COPD (59). Whether or not our observation of a different co-regulation of TL and mtDNA-CN in old patients with COPD relates to their higher incidence of lung cancer deserves further investigation (60).

### Strengths and Potential Limitations

To our knowledge, our study is the first to investigate two ageing hallmarks in young and old patients with COPD. Yet, we acknowledge several potential limitations. First, the sample size of the young patients with COPD and older controls is limited, and the cases and controls are not age-paired, accordingly, all the models were adjusted by age. Second, to avoid a potential confounding effect of tobacco smoking, controls were ever smokers with normal lung function and we did not study never smokers, which could have given us additional information. Finally, the blood cell counts were not available in our study so we could not include them as covariates in our models.

## Conclusions

Telomere length, but not mtDNA-CN, is associated with lung function parameters independently of age, sex, and smoking. Short telomeres are observed both in young and old patients with COPD with severe airflow limitation, suggesting that telomere attrition may be involved in some COPD endotypes and in the young patients. The different relationship between TL and mtDNA-CN in young and old patients with COPD deserves further investigation. Finally, more studies investigating several hallmarks of ageing are needed to better understand the influence of accelerated ageing in young patients with COPD.

## Data Availability Statement

The raw data supporting the conclusions of this article will be made available by the authors, without undue reservation.

## Ethics Statement

The studies involving human participants were reviewed and approved by Ethics Committee of Hospital Clinic (Barcelona, Spain). Study (HCB-2018/135). The patients/participants provided their written informed consent to participate in this study.

## Author Contributions

SC-R, RF, and AA: study conception and design. AA-C, AB-S, SP-G, JG, GG, NM, AL-G, TG, and BC: data acquisition. RF, AA, SC-R, and BC: data analysis. RF, AA, and SC-R: manuscript preparation. All authors: manuscript revision.

## Funding

This work was supported in part by the unrestricted grants from the Menarini, Instituto de Salud Carlos III (PI17/00379, PI18/00018), SEPAR (068/2015). RF is recipient of a Miguel Servet Research Program Contract (FEDER, CP16/00039), SC-R is recipient of a P-FIS (2018) grant.

## Conflict of Interest

The authors declare that the research was conducted in the absence of any commercial or financial relationships that could be construed as a potential conflict of interest.

## Publisher's Note

All claims expressed in this article are solely those of the authors and do not necessarily represent those of their affiliated organizations, or those of the publisher, the editors and the reviewers. Any product that may be evaluated in this article, or claim that may be made by its manufacturer, is not guaranteed or endorsed by the publisher.

## Appendix

Full list of field investigators of the study:

- Hospital Universitario Son Espases (Mallorca): Borja G. Cosio; Rocío Cordova Diaz; María Magdalena Pan Naranjo; Joan Palmer Sancho; Miguel Román Rodríguez;
- Hospital Clínic (Barcelona): Joan Albert Barberà; Josep Roca Torrent; Yolanda Torralba Garcia; JorgeMoises Lafuente; Anna Maria Pedro Pijoan; Amparo Hervas Docón; Carmen Herranz; Núria Sanchez Ruano;
- Hospita del Mar (Barcelona): Joaquim Gea; Diego A; Rodríguez Chiaradía; Anna Rodó-Pin; Clara Martín-Ontiyuelo; MireiaAdmetlló; Concepción Ballano Castro; Laura Gutiérrez Martín; José Ignacio Aoiz Linares; Sergi Pascual-Guardia; Marta Mourelo Cereijo.
- ISGLOBAL, Garcia-Aymerich J, Alicia Borras.
- Fundación Jiménez Díaz (Madrid): Germán Peces-Barba Romero; José Fernández Arias; Carolina Gotera Rivera; Manuel Martin Bernal; Guillermo Gallardo Madueño; Andrés Alcázar Peral; Carmelo Palacios Miras; Maria Teresa Pinedo Moraleda; Maria Belén Torres Labandeira; Mercedes Colomo Rodríguez; María Concepción Rodríguez Gallego; Carmen Lobon Agundez; Mónica Nácher Conches; María José Mansilla; Rosario Serrano Martín
- Hospital 12 Octubre (Madrid): Carlos J. Álvarez Martínez; Marta Padilla Bernáldez; Jesús Molina París
- Hospital ParcTaulí (Sabadell): Laura Vigil Giménez; Eduard Monsó Molas; Laia Seto Gort; Montserrat Baré Mañas; Anna Maria Fabra Noguera
- Hospital Virgen del Rocío (Sevilla): José Luís López Campos; Carmen Calero Acuña; Laura Carrasco Hernández
- Hospital Universitario de Bellvitge (Hospitalet de Llobregat; Barcelona): Salud Santos Perez; Montserrat Navarro; Elisabeth Serra; Ferran Ferrer Keysers; Damaris Batallé; M; Dolores Peleato Catalan; Albert Dorca; Javier Burgos
- Hospital Arnau de Vilanova (Valencia): Juan José Soler-Cataluña; Noelia González García; Lourdes Sánchez Sánchez
- Hospital Universitario Central de Asturias (Oviedo): Cristina Martínez González; Amador Prieto Fernández; Susana Martínez González
- Hospital Candelaria (Canarias): Ciro Casanova Macario; Delia Mayato
- Hospital Universitario de A Coruña: Pedro J Marcos Rodriguez; Luis Domínguez Juncal; Rosario Timiraos Carrasco; Rosa Garcia Palenzuela.
