# Supplementary material for: Telomere Length but Not Mitochondrial DNA Copy Number Is Altered in Both Young and Old COPD
Source: Front Med (Lausanne). 2021 Nov 24;8:761767. doi: 10.3389/fmed.2021.761767 (PMC8652089; doi:10.3389/fmed.2021.761767)
Supplement: Supplementary file 1 [file Data_Sheet_1.docx]

**TELOMERE LENGTH BUT NOT MITOCHONDRIAL DNA COPY NUMBER IS ALTERED IN BOTH YOUNG AND OLD COPD PATIENTS**

Sandra Casas-Recasens^1^, Nuria Mendoza^2^, Alejandra López-Giraldo ^1,2,3^, Tamara Garcia^2^, Borja G Cosio^1,4,5^, Sergi Pascual-Guardia^1,6,7^, Ady Acosta-Castro^1,8^, Alicia Borras-Santos^1,9^, Joaquim Gea^1,6,7^, Garrabou G^2,10,11,12^, Alvar Agusti^1,2,3,10^, Rosa Faner^1,2^ on behalf of all investigators of the EARLY COPD and BIOMEPOC projects (listed in the Appendix).

# SUPPLEMENTARY METHODS

**Characterization of participants**

Young participants (35–50 years of age) were characterized at 12 tertiary hospitals in Spain as previously described^8^. Recruitment was by local advertisement, and from an automatically generated list of smokers by affiliated primary care to tertiary hospitals. All participants were Caucasians, current or former smokers (>10 pack-years). Cases were defined by the presence of airflow limitation after bronchodilation (i.e. FEV1/FVC<0.7), and controls as FEV1/ FVC≥0.7. Exclusion criteria were: α1-antitrypsin deficiency, conditions that could potentially limit future follow-up (e.g. foreseen changes of residence, psychiatric diseases), chronic inflammatory or autoimmune diseases, severe bronchiectasis, active tuberculosis or cancer. Exclusion criteria for controls also included a previously confirmed diagnosis of asthma. All participants had been free of any acute respiratory condition for 8 weeks prior to the baseline study visit.

Old participants (>50 yrs.) were characterized at 7 tertiary hospitals in Spain as previously described^7^. Patients with COPD and a group of healthy controls were studied with continuous follow-up in a specialized respiratory medicine clinic for at least 2 years. Diagnostic criteria for COPD were a history of smoking, and a post-bronchodilator FEV1/ FVC<0.7. Exclusion criteria were: (1) diagnosis of another associated respiratory pathology that conditions care such as pulmonary fibrosis, kyphoscoliosis, obesity-hypoventilation, neuromuscular pathology, upper airway obstruction, significant bronchiectasis, extensive tuberculous sequelae, asthma, bronchiolitis or uncontrolled bronchogenic carcinoma; and (2) the presence of extra pulmonary pathology that conditions care such as significant heart disease with chronic heart failure, advanced dementia, widespread neoplasia, end-stage liver or kidney failure, or other situations in the opinion of the investigator.

**Case and control definition**

In our study we categorized as COPD those individuals with a FEV1/FVC<0.7 (fixed ratio). If an FEV1/FVC<LLN was used instead, results were by and large similar. Specifically, using the lower limit of normal (LLN) instead of a fixed FEV1/FVC <0.7 ratio in the <=50 yrs.: (1) one young control changed to COPD; (2) two young COPD patients changed to controls. In the old group, 24 old COPD patients were reclassified as controls accordingly to the FEV1/FVC <LLN.

**TL measurement**

TL was measured in DNA by quantitative PCR (qPCR) following the method described by Cawthon et al.(26). Briefly, the number of telomere repeats and that of albumin (a single copy gene) were assessed in the same qPCR tube. The PCR reaction included: 1x LigthCycler 480 SYBR Green master (Roche, Mannenheim, Germany), 5 ng of DNA, 1500 nmols of each telomere primer (telg: ﻿ACA CTA AGG TTT GGG TTT GGG TTT GGG TTT GGG TTA GTG T and telc: ﻿TGT TAG GTA TCC CTA TCC CTA TCC CTA TCC CTA TCC CTA ACA), and 100 nmols of each albumin primer (albu: ﻿CGG CGG CGG GCG GCG CGG GCT GGG CGG aaa tgc tgc aca gaa tcc ttg and albd: ﻿GCC CGG CCC GCC GCG CCC GTC CCG CCG gaa aag cat ggt cgc ctg tt). The cycling conditions were as follows: 15 min of denaturation at 95°C; priming: 2 cycles of 15 s at 94°C, 15 s at 49°C; and cycling: 32 cycles of 15 s at 94°C, 10 s at 62°C, 15 s at 74°C with signal acquisition, 10 s at 84°C, 15 s at 88°C with signal acquisition. In our setting, the 74°C reads provided the threshold cycle (C_t_) of the telomeres, while the 88°C reads provided the C_t_ of the albumin template. All reactions were run on a LightCycler 480 (Roche, Mannenheim, Germany) in 384-well plates, by quadruplicate, the C_t_ was de mean of the 3 out of 4 measurements with the lowest SD (maximum SD= 15%). The TL and albumin concentration was obtained using a standard curve, set up with eight serial dilutions of the DNA of a 27-year-old male, included in each run. TL is expressed as relative telomere to single copy gene ratio as described by Cawthon et al.(26).

**mtDNA-CN quantification**

To determine mtDNA-CN in DNA, the mitochondrial 12S ribosomal RNA (mt12SrRNA) gene and the nuclear-encoded *RNAse P* gene were simultaneously determined in the same qPCR tube, as previously described (28) The PCR reaction included: Taqman Universal Master Mix II, with UNG (Applied Biosystems, US), 3.125 ng of DNA, 1250 nmols of each 12s rRNA primer (12s rRNA_f: 5'CCA CGG GAA ACA GCA GCA GTG AT3'﻿ and 12s rRNA_r: ﻿5'CTA TTG ACT TGG GTT AAT CGT GTG A3'), 1250 nmols of Taqman probe 12s rRNA (6FAM-5'TGC CAG CCA CCG CG3' MGB) and 100 nmols of RNAsaP Control Reagent (VIC) including the primers and VIC labelled Taqman probe for RNAsaP (Applied Biosystems, US). The cycling conditions were as follows: 2min at 50°C, 10 min at 95°C; and cycling: 40 cycles of 15 s at 95°C, 60 s at 60°C. All reactions were run on a qPCR in ViiA 7 (Real-Time PCR System, Applied Biosystems) in 384-well plates, by triplicates, the C_t_ was de mean of the 2 out of 3 measurements with the lowest SD (maximum SD= 15%). The concentration was obtained using a standard curve, in turn obtained by serial dilutions of the plasmids containing RNAseP and 12SrRNA inserts, as previously described(28). The mtDNA-CN was calculated as the ratio 12SrRNA mtDNA copies / RNAseP DNA copies.

***SUPPLEMENTARY TABLES:***

**Table S1.** β estimates (regression coefficients) and 95% CI from the multivariate analysis of the association between age, lung function parameters (FEV1/FVC%, FEV1% ref., DLCO% ref.), severity of airflow limitation and emphysema with telomere length (log(TL)) as the outcome and the other variables as covariates in the total pooled population, in young COPD patients and in old COPD patients.

| **Age vs log(TL) in all individuals** | | | | | | | |
| --- | --- | --- | --- | --- | --- | --- | --- |
| **Variable** | **level** | **n** | **log(TL) estimate** | **std.error** | **p.value** | **conf.low** | **conf.high** |
| **age** |  | 393 | -8.47E-01 | 0.00109 | 5.88E-14 | -1.06E-02 | -6.33E-03 |
| sex | F | 137 | Reference |  |  |  |  |
| sex | M | 256 | -6.38E-01 | 0.0224 | 4.60e- | 3.00E+00 | -1.08E-01 |
| smoking | CS | 256 | Reference |  |  |  |  |
| smoking | FS | 137 | -2.70E-01 | 0.0227 | 3.62E-01 | -6.54E-02 | 2.39E-02 |
| pack_year |  | 393 | -1.20E-01 | 0.000538 | 5.84E-02 | -2.08E-03 | 3.66E-05 |
| **FEV1/FVC% ratio on Telomere length in all individuals** | | | | | | | |
| **Variable** | **level** | **n** | **log(TL) estimate** | **std.error** | **p.value** | **conf.low** | **conf.high** |
| **FEV_1_/FVC%** |  | 435 | 3.20E-01 | 0.000731 | 4.36E-05 | 1.58E-03 | 4.46E-03 |
| age |  | 435 | -7.55E-01 | 0.001 | 3.00E-13 | -9.53E-03 | -5.58E-03 |
| sex | F | 147 | Reference |  |  |  |  |
| sex | M | 288 | -6.20E-01 | 0.0216 | 4.26E-03 | -1.04E-01 | -1.96E-02 |
| **FEV1/FVC% ratio on Telomere length in controls** | | | | | | | |
| **Variable** | **level** | **n** | **log(TL) estimate** | **std.error** | **p.value** | **conf.low** | **conf.high** |
| **FEV_1_/FVC%** |  | 195 | 2.78E-01 | 0.00266 | 2.98E-01 | -2.47E-03 | 8.03E-03 |
| age |  | 195 | -8.55E-01 | 0.00185 | 7.22E-06 | -1.22E-02 | -4.90E-03 |
| sex | F | 95 | Reference |  |  |  |  |
| sex | M | 100 | -6.51E-01 | 0.028 | 2.11E-02 | -1.20E-01 | -9.87E-03 |
| **FEV1/FVC% ratio on Telomere length in COPD patients** | | | | | | | |
| **Variable** | **level** | **n** | **log(TL) estimate** | **std.error** | **p.value** | **conf.low** | **conf.high** |
| **FEV_1_/FVC%** |  | 240 | 3.58E-01 | 0.00112 | 1.63E-03 | 1.37E-03 | 5.79E-03 |
| age |  | 240 | -7.36E-01 | 0.00128 | 2.77E-08 | -9.88E-03 | -4.84E-03 |
| sex | F | 52 | Reference |  |  |  |  |
| sex | M | 188 | -6.33E-01 | 0.034 | 6.41E-02 | -1.30E-01 | 3.73E-03 |
| **FEV1/FVC% ratio on Telomere length in young COPD patients** | | | | | | | |
| **Variable** | **level** | **n** | **log(TL) estimate** | **std.error** | **p.value** | **conf.low** | **conf.high** |
| **FEV_1_/FVC%** |  | 81 | 3.17E-01 | 0.00217 | 1.48E-01 | -1.15E-03 | 7.49E-03 |
| age |  | 81 | -4.00E-01 | 0.00655 | 5.43E-01 | -1.70E-02 | 9.04E-03 |
| sex | F | 26 | Reference |  |  |  |  |
| sex | M | 55 | -5.63E-01 | 0.0488 | 2.52E-01 | -1.53E-01 | 4.08E-02 |
| **FEV1/FVC% ratio on Telomere length in old COPD patients** | | | | | | | |
| **Variable** | **level** | **n** | **log(TL) estimate** | **std.error** | **p.value** | **conf.low** | **conf.high** |
| **FEV_1_/FVC%** |  | 159 | 3.25E-01 | 0.00137 | 1.87E-02 | 5.49E-04 | 5.95E-03 |
| age |  | 159 | -4.69E-01 | 0.00233 | 4.56E-02 | -9.29E-03 | -9.40E-05 |
| sex | F | 26 | Reference |  |  |  |  |
| sex | M | 133 | -7.55E-01 | 0.0486 | 1.22E-01 | -1.72E-01 | 2.05E-02 |
| **FEV1% ref. on Telomere length in all individuals** | | | | | | | |
| **Variable** | **level** | **n** | **log(TL) estimate** | **std.error** | **p.value** | **conf.low** | **conf.high** |
| **FEV_1_% ref.** |  | 434 | 2.80E-01 | 0.000425 | 1.45E-06 | 1.24E-03 | 2.91E-03 |
| age |  | 434 | -7.23E-01 | 0.000989 | 1.29E-12 | -9.17E-03 | -5.29E-03 |
| sex | F | 146 | Reference |  |  |  |  |
| sex | M | 288 | -6.17E-01 | 0.0215 | 4.22E-03 | -1.04E-01 | -1.95E-02 |
| **FEV1% ref. on Telomere length in controls** | | | | | | | |
| **Variable** | **level** | **n** | **log(TL) estimate** | **std.error** | **p.value** | **conf.low** | **conf.high** |
| **FEV_1_% ref.** |  | 194 | 5.45E-01 | 0.00103 | 5.97E-01 | -1.48E-03 | 2.57E-03 |
| age |  | 194 | -8.86E-01 | 0.0018 | 1.92E-06 | -1.24E-02 | -5.30E-03 |
| sex | F | 94 | Reference |  |  |  |  |
| sex | M | 100 | -6.77E-01 | 0.0282 | 1.75E-02 | -1.23E-01 | -1.20E-02 |
| **FEV1% ref. on Telomere length in COPD patients** | | | | | | | |
| **Variable** | **level** | **n** | **log(TL) estimate** | **std.error** | **p.value** | **conf.low** | **conf.high** |
| **FEV_1_% ref.** |  | 240 | 2.83E-01 | 0.000616 | 7.05E-06 | 1.62E-03 | 4.05E-03 |
| age |  | 240 | -6.37E-01 | 0.00129 | 1.44E-06 | -8.91E-03 | -3.83E-03 |
| sex | F | 52 | Reference |  |  |  |  |
| sex | M | 188 | -6.65E-01 | 0.0332 | 4.66E-02 | -1.32E-01 | -1.02E-03 |
| **FEV1% ref. on Telomere length in young COPD patients** | | | | | | | |
| **Variable** | **level** | **n** | **log(TL) estimate** | **std.error** | **p.value** | **conf.low** | **conf.high** |
| **FEV_1_% ref.** |  | 81 | 1.70E-01 | 0.00107 | 1.18E-01 | -4.40E-04 | 3.84E-03 |
| age |  | 81 | -3.27E-01 | 0.00659 | 6.21E-01 | -1.64E-02 | 9.85E-03 |
| sex | F | 26 | Reference |  |  |  |  |
| sex | M | 55 | -6.23E-01 | 0.0487 | 2.05E-01 | -1.59E-01 | 3.48E-02 |
| **FEV1% ref. on Telomere length in old COPD patients** | | | | | | | |
| **Variable** | **level** | **n** | **log(TL) estimate** | **std.error** | **p.value** | **conf.low** | **conf.high** |
| **FEV_1_% ref.** |  | 159 | 3.21E-01 | 0.000797 | 8.83E-05 | 1.63E-03 | 4.78E-03 |
| age |  | 159 | -4.74E-01 | 0.00225 | 3.69E-02 | -9.19E-03 | -2.93E-04 |
| sex | F | 26 |  |  |  |  |  |
| sex | M | 133 | -6.49E-01 | 0.0471 | 1.71E-01 | -1.58E-01 | 2.82E-02 |
| **DLCO% ref. on Telomere length in all individuals** | | | | | | | |
| **Variable** | **level** | **n** | **log(TL) estimate** | **std.error** | **p.value** | **conf.low** | **conf.high** |
| **DLCO% ref.** |  | 349 | 1.62E-01 | 0.000448 | 3.35E-04 | 7.43E-04 | 2.51E-03 |
| age |  | 349 | -7.46E-01 | 0.00113 | 1.77E-10 | -9.68E-03 | -5.23E-03 |
| sex | F | 126 | Reference |  |  |  |  |
| sex | M | 223 | -6.96E-01 | 0.0237 | 3.50E-03 | -1.16E-01 | -2.30E-02 |
| **DLCO% ref. on Telomere length in controls** | | | | | | | |
| **Variable** | **level** | **n** | **log(TL) estimate** | **std.error** | **p.value** | **conf.low** | **conf.high** |
| **DLCO% ref.** |  | 169 | 1.63E-01 | 0.00106 | 1.25E-01 | -4.59E-04 | 3.71E-03 |
| age |  | 169 | -9.43E-01 | 0.00196 | 3.52E-06 | -1.33E-02 | -5.55E-03 |
| sex | F | 84 | Reference |  |  |  |  |
| sex | M | 85 | -6.12E-01 | 0.03 | 4.32E-02 | -1.21E-01 | -1.88E-03 |
| **DLCO% ref. on Telomere length in COPD patients** | | | | | | | |
| **Variable** | **level** | **n** | **log(TL) estimate** | **std.error** | **p.value** | **conf.low** | **conf.high** |
| **DLCO% ref.** |  | 180 | 1.75E-01 | 0.000593 | 3.63E-03 | 5.79E-04 | 2.92E-03 |
| age |  | 180 | -5.66E-01 | 0.00166 | 8.23E-04 | -8.95E-03 | -2.38E-03 |
| sex | F | 42 | Reference |  |  |  |  |
| sex | M | 138 | -7.97E-01 | 0.0391 | 4.30E-02 | -1.57E-01 | -2.52E-03 |
| **DLCO% ref. on Telomere length in young COPD patients** | | | | | | | |
| **Variable** | **level** | **n** | **log(TL) estimate** | **std.error** | **p.value** | **conf.low** | **conf.high** |
| **DLCO% ref.** |  | 72 | 1.45E-01 | 0.00113 | 2.05E-01 | -8.11E-04 | 3.72E-03 |
| age |  | 72 | -1.80E-01 | 0.00733 | 8.06E-01 | -1.64E-02 | 1.28E-02 |
| sex | F | 23 | Reference |  |  |  |  |
| sex | M | 49 | -8.22E-01 | 0.0553 | 1.42E-01 | -1.92E-01 | 2.82E-02 |
| **DLCO% ref. on Telomere length in old COPD patients** | | | | | | | |
| **Variable** | **level** | **n** | **log(TL) estimate** | **std.error** | **p.value** | **conf.low** | **conf.high** |
| **DLCO% ref.** |  | 108 | 1.76E-01 | 0.000754 | 2.16E-02 | 2.64E-04 | 3.26E-03 |
| age |  | 108 | -4.74E-01 | 0.00303 | 1.21E-01 | -1.07E-02 | 1.27E-03 |
| sex | F | 19 | Reference |  |  |  |  |
| sex | M | 89 | -7.28E-01 | 0.0578 | 2.11E-01 | -1.87E-01 | 4.18E-02 |
| **FEV1<50% vs. FEV1>50% vs Telomere length in young COPD patients** | | | | | | | |
| **Variable** | **level** | **n** | **log(TL) estimate** | **std.error** | **p.value** | **conf.low** | **conf.high** |
| **severity** | FEV1% ≥ 50 | 66 | Reference |  |  |  |  |
| **severity** | FEV1% < 50 | 15 | -1.43E-01 | 0.0567 | 1.35E-02 | -2.56E-01 | -3.04E-02 |
| age |  | 81 | -5.80E-01 | 0.00634 | 4.25E-01 | -1.77E-02 | 7.55E-03 |
| sex | F | 26 | Reference |  |  |  |  |
| sex | M | 55 | -5.91E-01 | 0.0475 | 2.17E-01 | -1.54E-01 | 3.55E-02 |
| **FEV1<50% vs. FEV1>50% vs Telomere length in old COPD patients** | | | | | | | |
| **Variable** | **level** | **n** | **log(TL) estimate** | **std.error** | **p.value** | **conf.low** | **conf.high** |
| **severity** | FEV1% ≥ 50 | 77 | Reference |  |  |  |  |
| **severity** | FEV1% < 50 | 82 | -1.44E-01 | 0.0325 | 1.71E-05 | -2.08E-01 | -8.01E-02 |
| age |  | 159 | -4.94E-01 | 0.00223 | 2.84E-02 | -9.35E-03 | -5.28E-04 |
| sex | F | 26 | Reference |  |  |  |  |
| sex | M | 133 | -7.71E-01 | 0.0463 | 9.80E-02 | -1.69E-01 | 1.44E-02 |
| **emphysema (DLCO<60 vs. >80) on Telomere length in young COPD patients** | | | | | | | |
| **Variable** | **level** | **n** | **log(TL) estimate** | **std.error** | **p.value** | **conf.low** | **conf.high** |
| **Emphysema** | No | 44 | Reference |  |  |  |  |
| **Emphysema** | Yes | 11 | -8.98E-01 | 0.0693 | 2.01E-01 | -2.29E-01 | 4.93E-02 |
| age |  | 55 | 2.66E-01 | 0.00729 | 7.17E-01 | -1.20E-02 | 1.73E-02 |
| sex | F | 15 | Reference |  |  |  |  |
| sex | M | 40 | -3.33E-01 | 0.0616 | 5.91E-01 | -1.57E-01 | 9.03E-02 |
| **emphysema (DLCO<60 vs. >80) on Telomere length in old COPD patients** | | | | | | | |
| **Variable** | **level** | **n** | **log(TL) estimate** | **std.error** | **p.value** | **conf.low** | **conf.high** |
| **Emphysema** | No | 16 | Reference |  |  |  |  |
| **Emphysema** | Yes | 62 | -1.46E-01 | 0.0617 | 2.02E-02 | -2.69E-01 | -2.35E-02 |
| age |  | 78 | -4.12E-01 | 0.0036 | 2.57E-01 | -1.13E-02 | 3.06E-03 |
| sex | F | 11 | Reference |  |  |  |  |
| sex | M | 67 | -7.54E-01 | 0.076 | 3.25E-01 | -2.27E-01 | 7.61E-02 |

**Table S2**. β estimates (regression coefficients) and 95% CI from the multivariate analysis of the association between age, lung function parameters (FEV1/FVC%, FEV1% ref., DLCO% ref.), severity of airflow limitation and emphysema with mtDNA copy number (log(mtDNA-CN)) as the outcome and the other variables as covariates in the total pooled population, in young COPD patients and in old COPD patients.

| **Age vs mtDNA-CN in all individuals** | | | | | | | |
| --- | --- | --- | --- | --- | --- | --- | --- |
| **Variable** | **level** | **n** | **estimate** | **std.error** | **p.value** | **conf.low** | **conf.high** |
| **age** |  | 393 | -1.76E-02 | 0.00256 | 2.46E-11 | -2.26E-02 | -1.26E-02 |
| sex | F | 137 | Reference |  |  |  |  |
| sex | M | 256 | -4.21E-02 | 0.0527 | 4.25E-01 | -1.46E-01 | 6.15E-02 |
| smoking | CS | 256 | Reference |  |  |  |  |
| smoking | FS | 137 | 7.50E-02 | 0.0535 | 1.62E-01 | -3.01E-02 | 1.80E-01 |
| pack_year |  | 393 | -4.17E-04 | 0.00127 | 7.42E-01 | -2.91E-03 | 2.07E-03 |
| **FEV1/FVC% ratio on mtDNA-CN in all individuals** | | | | | | | |
| **Variable** | **level** | **n** | **estimate** | **std.error** | **p.value** | **conf.low** | **conf.high** |
| **FEV**_1_/FVC% |  | 435 | 6.40E-04 | 0.00175 | 7.15E-01 | -2.81E-03 | 4.09E-03 |
| age |  | 435 | -1.57E-02 | 0.00241 | 2.16E-10 | -2.04E-02 | -1.09E-02 |
| sex | F | 147 | Reference |  |  |  |  |
| sex | M | 288 | -3.06E-02 | 0.0518 | 5.55E-01 | -1.32E-01 | 7.12E-02 |
| **FEV1/FVC% ratio on mtDNA-CN in controls** | | | | | | | |
| **Variable** | **level** | **n** | **estimate** | **std.error** | **p.value** | **conf.low** | **conf.high** |
| **FEV**_1_/FVC% |  | 195 | 2.02E-02 | 0.00687 | 3.74E-03 | 6.62E-03 | 3.37E-02 |
| age |  | 195 | -1.28E-02 | 0.00478 | 8.20E-03 | -2.22E-02 | -3.34E-03 |
| sex | F | 95 | Reference |  |  |  |  |
| sex | M | 100 | 1.51E-02 | 0.0722 | 8.34E-01 | -1.27E-01 | 1.58E-01 |
| **FEV1/FVC% ratio on mtDNA-CN in COPD patients** | | | | | | | |
| **Variable** | **level** | **n** | **estimate** | **std.error** | **p.value** | **conf.low** | **conf.high** |
| **FEV**_1_/FVC% |  | 240 | -5.28E-04 | 0.00249 | 8.32E-01 | -5.44E-03 | 4.38E-03 |
| age |  | 240 | -1.64E-02 | 0.00284 | 2.42E-08 | -2.20E-02 | -1.08E-02 |
| sex | F | 52 | Reference |  |  |  |  |
| sex | M | 188 | -7.93E-02 | 0.0754 | 2.94E-01 | -2.28E-01 | 6.93E-02 |
| **FEV1/FVC% ratio on mtDNA-CN in young COPD patients** | | | | | | | |
| **Variable** | **level** | **n** | **estimate** | **std.error** | **p.value** | **conf.low** | **conf.high** |
| **FEV**_1_/FVC% |  | 81 | 2.09E-03 | 0.00678 | 7.59E-01 | -1.14E-02 | 1.56E-02 |
| age |  | 81 | -3.48E-03 | 0.0205 | 8.65E-01 | -4.42E-02 | 3.73E-02 |
| sex | F | 26 | Reference |  |  |  |  |
| sex | M | 55 | -9.17E-02 | 0.152 | 5.49E-01 | -3.95E-01 | 2.12E-01 |
| **FEV1/FVC% ratio on mtDNA-CN in old COPD patients** | | | | | | | |
| **Variable** | **level** | **n** | **estimate** | **std.error** | **p.value** | **conf.low** | **conf.high** |
| **FEV**_1_/FVC% |  | 159 | -2.07E-03 | 0.00226 | 3.60E-01 | -6.53E-03 | 2.39E-03 |
| age |  | 159 | -1.30E-02 | 0.00385 | 9.23E-04 | -2.06E-02 | -5.40E-03 |
| sex | F | 26 | 0.00E+00 |  |  |  |  |
| sex | M | 133 | -7.12E-02 | 0.0803 | 3.76E-01 | -2.30E-01 | 8.74E-02 |
| **FEV1% ref. on mtDNA-CN in all individuals** | | | | | | | |
| **Variable** | **level** | **n** | **estimate** | **std.error** | **p.value** | **conf.low** | **conf.high** |
| **FEV**_1_% ref. |  | 434 | 8.10E-05 | 0.00103 | 9.37E-01 | -1.94E-03 | 2.10E-03 |
| age |  | 434 | -1.60E-02 | 0.00239 | 7.59E-11 | -2.07E-02 | -1.13E-02 |
| sex | F | 146 | Reference |  |  |  |  |
| sex | M | 288 | -3.52E-02 | 0.0519 | 4.98E-01 | -1.37E-01 | 6.67E-02 |
| **FEV1% ref. on mtDNA-CN in controls** | | | | | | | |
| **Variable** | **level** | **n** | **estimate** | **std.error** | **p.value** | **conf.low** | **conf.high** |
| **FEV**_1_% ref. |  | 194 | 1.76E-03 | 0.00271 | 5.15E-01 | -3.57E-03 | 7.10E-03 |
| age |  | 194 | -1.62E-02 | 0.00474 | 7.58E-04 | -2.56E-02 | -6.88E-03 |
| sex | F | 94 | Reference |  |  |  |  |
| sex | M | 100 | -2.29E-03 | 0.0744 | 9.75E-01 | -1.49E-01 | 1.44E-01 |
| **FEV1% ref. on mtDNA-CN in COPD patients** | | | | | | | |
| **Variable** | **level** | **n** | **estimate** | **std.error** | **p.value** | **conf.low** | **conf.high** |
| **FEV**_1_% ref. |  | 240 | 1.07E-04 | 0.0014 | 9.39E-01 | -2.65E-03 | 2.86E-03 |
| age |  | 240 | -1.62E-02 | 0.00292 | 7.89E-08 | -2.19E-02 | -1.04E-02 |
| sex | F | 52 | Reference |  |  |  |  |
| sex | M | 188 | -7.81E-02 | 0.0753 | 3.01E-01 | -2.27E-01 | 7.02E-02 |
| **FEV1% ref. on mtDNA-CN in young COPD patients** | | | | | | | |
| **Variable** | **level** | **n** | **estimate** | **std.error** | **p.value** | **conf.low** | **conf.high** |
| **FEV**_1_% ref. |  | 81 | 2.13E-03 | 0.00336 | 5.29E-01 | -4.57E-03 | 8.82E-03 |
| age |  | 81 | -1.96E-03 | 0.0206 | 9.24E-01 | -4.30E-02 | 3.91E-02 |
| sex | F | 26 | Reference |  |  |  |  |
| sex | M | 55 | -9.85E-02 | 0.152 | 5.20E-01 | -4.02E-01 | 2.05E-01 |
| **FEV1% ref. on mtDNA-CN in old COPD patients** | | | | | | | |
| **Variable** | **level** | **n** | **estimate** | **std.error** | **p.value** | **conf.low** | **conf.high** |
| **FEV**_1_% ref. |  | 159 | -1.34E-03 | 0.00136 | 3.26E-01 | -4.02E-03 | 1.34E-03 |
| age |  | 159 | -1.31E-02 | 0.00384 | 8.57E-04 | -2.06E-02 | -5.47E-03 |
| sex | F | 26 | Reference |  |  |  |  |
| sex | M | 133 | -7.23E-02 | 0.0803 | 3.69E-01 | -2.31E-01 | 8.63E-02 |
| **DLCO% ref. on mtDNA-CN in all individuals** | | | | | | | |
| **Variable** | **level** | **n** | **estimate** | **std.error** | **p.value** | **conf.low** | **conf.high** |
| **DLCO% ref.** |  | 349 | 9.97E-04 | 0.00109 | 3.62E-01 | -1.15E-03 | 3.15E-03 |
| age |  | 349 | -1.45E-02 | 0.00276 | 2.72E-07 | -1.99E-02 | -9.06E-03 |
| sex | F | 126 | Reference |  |  |  |  |
| sex | M | 223 | -2.89E-02 | 0.0577 | 6.17E-01 | -1.42E-01 | 8.47E-02 |
| **DLCO% ref. on mtDNA-CN in controls** | | | | | | | |
| **Variable** | **level** | **n** | **estimate** | **std.error** | **p.value** | **conf.low** | **conf.high** |
| **DLCO% ref.** |  | 169 | 5.11E-03 | 0.00273 | 6.31E-02 | -2.83E-04 | 1.05E-02 |
| age |  | 169 | -1.91E-02 | 0.00507 | 2.38E-04 | -2.91E-02 | -9.04E-03 |
| sex | Effect of FEV1 on mtDNA-CN+B109:J143 | 84 | Reference |  |  |  |  |
| sex | M | 85 | 6.88E-03 | 0.0776 | 9.29E-01 | -1.46E-01 | 1.60E-01 |
| **DLCO% ref. on mtDNA-CN in COPD patients** | | | | | | | |
| **Variable** | **level** | **n** | **estimate** | **std.error** | **p.value** | **conf.low** | **conf.high** |
| **DLCO% ref.** |  | 180 | 1.23E-03 | 0.00136 | 3.67E-01 | -1.46E-03 | 3.92E-03 |
| age |  | 180 | -1.58E-02 | 0.00382 | 5.27E-05 | -2.34E-02 | -8.29E-03 |
| sex | F | 42 | Reference |  |  |  |  |
| sex | M | 138 | -1.13E-01 | 0.0897 | 2.09E-01 | -2.90E-01 | 6.39E-02 |
| **DLCO% ref. on mtDNA-CN in young COPD patients** | | | | | | | |
| **Variable** | **level** | **n** | **estimate** | **std.error** | **p.value** | **conf.low** | **conf.high** |
| **DLCO% ref.** |  | 72 | 3.32E-03 | 0.00349 | 3.45E-01 | -3.64E-03 | 1.03E-02 |
| age |  | 72 | 1.42E-03 | 0.0225 | 9.50E-01 | -4.35E-02 | 4.64E-02 |
| sex | F | 23 | Reference |  |  |  |  |
| sex | M | 49 | -1.21E-01 | 0.17 | 4.78E-01 | -4.60E-01 | 2.18E-01 |
| **DLCO% ref. on mtDNA-CN in old COPD patients** | | | | | | | |
| **Variable** | **level** | **n** | **estimate** | **std.error** | **p.value** | **conf.low** | **conf.high** |
| **DLCO% ref.** |  | 108 | 3.08E-04 | 0.00121 | 8.00E-01 | -2.09E-03 | 2.71E-03 |
| age |  | 108 | -1.60E-02 | 0.00486 | 1.34E-03 | -2.57E-02 | -6.38E-03 |
| sex | F | 19 | Reference |  |  |  |  |
| sex | M | 89 | -1.10E-01 | 0.0928 | 2.37E-01 | -2.94E-01 | 7.36E-02 |
| **FEV1<50% vs. FEV1>50% vs mtDNA-CN in young COPD patients** | | | | | | | |
| **Variable** | **level** | **n** | **estimate** | **std.error** | **p.value** | **conf.low** | **conf.high** |
| **severity** | mild | 66 | Reference |  |  |  |  |
| **severity** | severe | 15 | -1.02E-01 | 0.182 | 5.76E-01 | -4.63E-01 | 2.60E-01 |
| age |  | 81 | -4.20E-03 | 0.0203 | 8.37E-01 | -4.47E-02 | 3.63E-02 |
| sex | F | 26 | Reference |  |  |  |  |
| sex | M | 55 | -9.36E-02 | 0.152 | 5.40E-01 | -3.97E-01 | 2.09E-01 |
| **FEV1<50% vs. FEV1>50% vs mtDNA-CN in old COPD patients** | | | | | | | |
| **Variable** | **level** | **n** | **estimate** | **std.error** | **p.value** | **conf.low** | **conf.high** |
| **severity** | mild | 77 | Reference |  |  |  |  |
| **severity** | severe | 82 | 5.78E-02 | 0.0559 | 3.03E-01 | -5.27E-02 | 1.68E-01 |
| age |  | 159 | -1.30E-02 | 0.00384 | 9.18E-04 | -2.06E-02 | -5.40E-03 |
| sex | F | 26 | Reference |  |  |  |  |
| sex | M | 133 | -6.70E-02 | 0.0797 | 4.02E-01 | -2.24E-01 | 9.04E-02 |
| **emphysema (DLCO<60 vs. >80) on mtDNA-CN in young COPD patients** | | | | | | | |
| **Variable** | **level** | **n** | **estimate** | **std.error** | **p.value** | **conf.low** | **conf.high** |
| **Emphysema** | No | 44 | Reference |  |  |  |  |
| **Emphysema** | Yes | 11 | -1.07E-01 | 0.245 | 6.66E-01 | -5.99E-01 | 3.86E-01 |
| age |  | 55 | 6.27E-03 | 0.0258 | 8.09E-01 | -4.55E-02 | 5.81E-02 |
| sex | F | 15 | Reference |  |  |  |  |
| sex | M | 40 | -3.29E-02 | 0.218 | 8.80E-01 | -4.70E-01 | 4.04E-01 |
| **emphysema (DLCO<60 vs. >80) on mtDNA-CN in old COPD patients** | | | | | | | |
| **Variable** | **level** | **n** | **estimate** | **std.error** | **p.value** | **conf.low** | **conf.high** |
| **Emphysema** | No | 16 | Reference |  |  |  |  |
| **Emphysema** | Yes | 62 | -3.06E-02 | 0.097 | 7.53E-01 | -2.24E-01 | 1.63E-01 |
| age |  | 78 | -1.53E-02 | 0.00567 | 8.61E-03 | -2.66E-02 | -4.00E-03 |
| sex | F | 11 | Reference |  |  |  |  |
| sex | M | 67 | -1.93E-02 | 0.12 | 8.72E-01 | -2.58E-01 | 2.19E-01 |

**Table S3.** β estimates (regression coefficients) and 95% CI from the multivariate analysis of the association between mtDNA copy number (log(mtDNA-CN)) with Telomere length (log(TL)) as the outcome and the other variables as covariates in young COPD patients and in old COPD patients.

| **log(mtDNA-CN) and log(TL) in young COPD patients** | | | | | | | |
| --- | --- | --- | --- | --- | --- | --- | --- |
| **Variable** | **level** | **n** | **log(TL) estimate** | **std.error** | **p.value** | **conf.low** | **conf.high** |
| **log(mtDNA)** |  | 81 | 1.51E-01 | 0.0327 | 1.42E-05 | 8.64E-02 | 2.16E-01 |
| age | age | 81 | -4.39E-03 | 0.00584 | 4.54E-01 | -1.60E-02 | 7.24E-03 |
| sex | F | 26 | Reference |  |  |  |  |
| sex | M | 55 | -4.35E-02 | 0.0438 | 3.23E-01 | -1.31E-01 | 4.37E-02 |
| **log(mtDNA-CN) and log(TL) in old COPD patients** | | | | | | | |
| **Variable** | **level** | **n** | **log(TL) estimate** | **std.error** | **p.value** | **conf.low** | **conf.high** |
| **log(mtDNA)** |  | 159 | -1.21E-01 | 0.0484 | 1.38E-02 | -2.16E-01 | -2.50E-02 |
| age |  | 159 | -5.91E-03 | 0.00241 | 1.51E-02 | -1.07E-02 | -1.16E-03 |
| sex | F | 26 | Reference |  |  |  |  |
| sex | M | 133 | -9.80E-02 | 0.0482 | 4.38E-02 | -1.93E-01 | -2.78E-03 |

**SUPPLEMENTARY FIGURES**

**ON-LINE FIGURES**

**Figure S1:** Association between Telomere Length (log(TL)) and FEV_1_/FVC. For this analysis, controls and COPD patients are defined as FEV_1_/FVC≥LLN or FEV_1_/FVC<LLN respectively. Blue dots identify COPD patients (n=208) whereas green dots correspond to controls (n=202).

**Figure S2**: Relationship between Telomere Length (log(mtDNA-CN)) and FEV_1_/FVC (Panel A), FEV_1_% ref. (Panel B) and DLCO% ref. (Panel C). Blue dots identify COPD patients (A: n=240, B: n=240, C: n=180) whereas green dots correspond to controls (A: n=195, B: n=194, C: n=169). Forest plots of the linear regression models of all individuals (A: n=435, B: n=434, C: n=349) presenting the point estimates and 95%CI (whiskers) of the change in log(mtDNA-CN) when adjusted for potential confounders. For further explanations, see text.

**Figure S3**: Relationship between Telomere Length (log(mtDNA-CN)) and FEV_1_/FVC (Panels A and D), FEV_1_% ref. (Panels B and E) and DLCO% ref. (Panels C and F in young (left) (A: n=81, B: n=81, C: n=72) and old (right) COPD patients (A: n=159, B: n=159, C: n=108). Forest plots of each linear regression model presenting the point estimates and 95%CI (whiskers) of the change in log(mtDNA-CN) when adjusted for potential confounders. For further explanations, see text.

**Figure S4**: (Panel A) Box plot of log(mtDNA-CN) by severity of airflow limitation in COPD patients stratified by young (n=81) and old (n=159), and corresponding Forest plots (bottom) of the linear regression model estimates presenting the point estimates and 95%CI (whiskers) of the change in log(mtDNA-CN) when adjusted for potential confounders. (Panel B) Box plot of log(mtDNA-CN) according to the presence (DLCO % ref. <60%) or absence (DLCO % ref. >80%) of emphysema in COPD patients stratified by young (n=55) and old (n=78), and corresponding Forest plots (bottom) of the linear regression model estimates presenting the point estimates and 95%CI (whiskers) of the change in log(mtDNA-CN) when adjusted for potential confounders.

Figure S5. Panel A: Scatter plot of log(TL) *vs.* log(mtDNA-CN) in young (n=157) (dark blue dots) and old (n=29) (red dots) controls excluding individuals with a mtDNA-CN > 2 standard deviations (SD) of the mean. Panel B: Scatter plot of log(TL) *vs.* log(mtDNA-CN) in young (n=75) (dark blue dots) and old (n=159) (red dots) COPD patients excluding individuals with a mtDNA-CN > 2 standard deviations (SD) of the mean.
