# Supplementary figures and images for: Telomere Length but Not Mitochondrial DNA Copy Number Is Altered in Both Young and Old COPD
Source: Front Med (Lausanne). 2021 Nov 24;8:761767. doi: 10.3389/fmed.2021.761767 (PMC8652089; doi:10.3389/fmed.2021.761767)

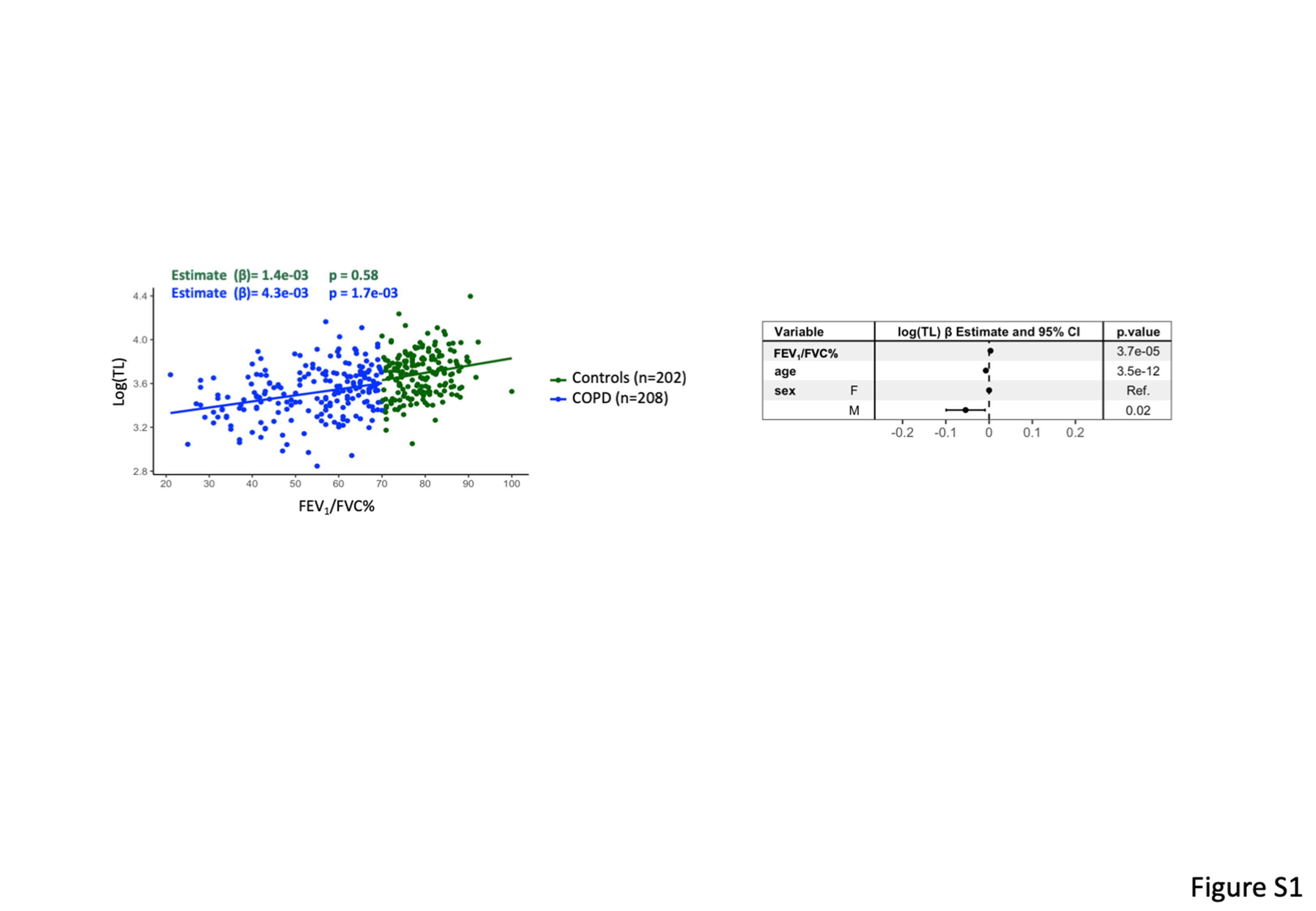

Supplement: Supplementary file 2 [file Image_1.TIFF]

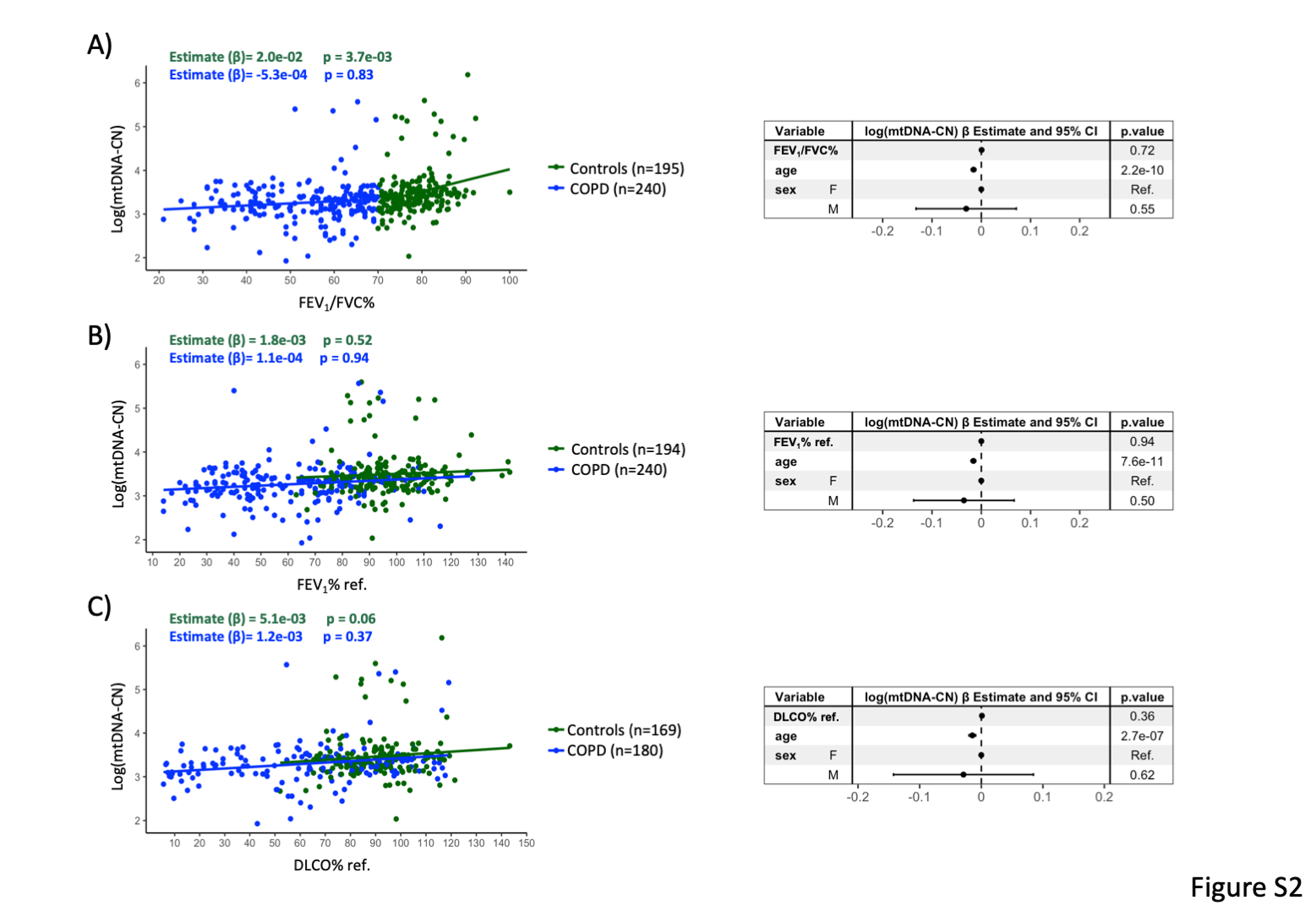

Supplement: Supplementary file 3 [file Image_2.TIFF]

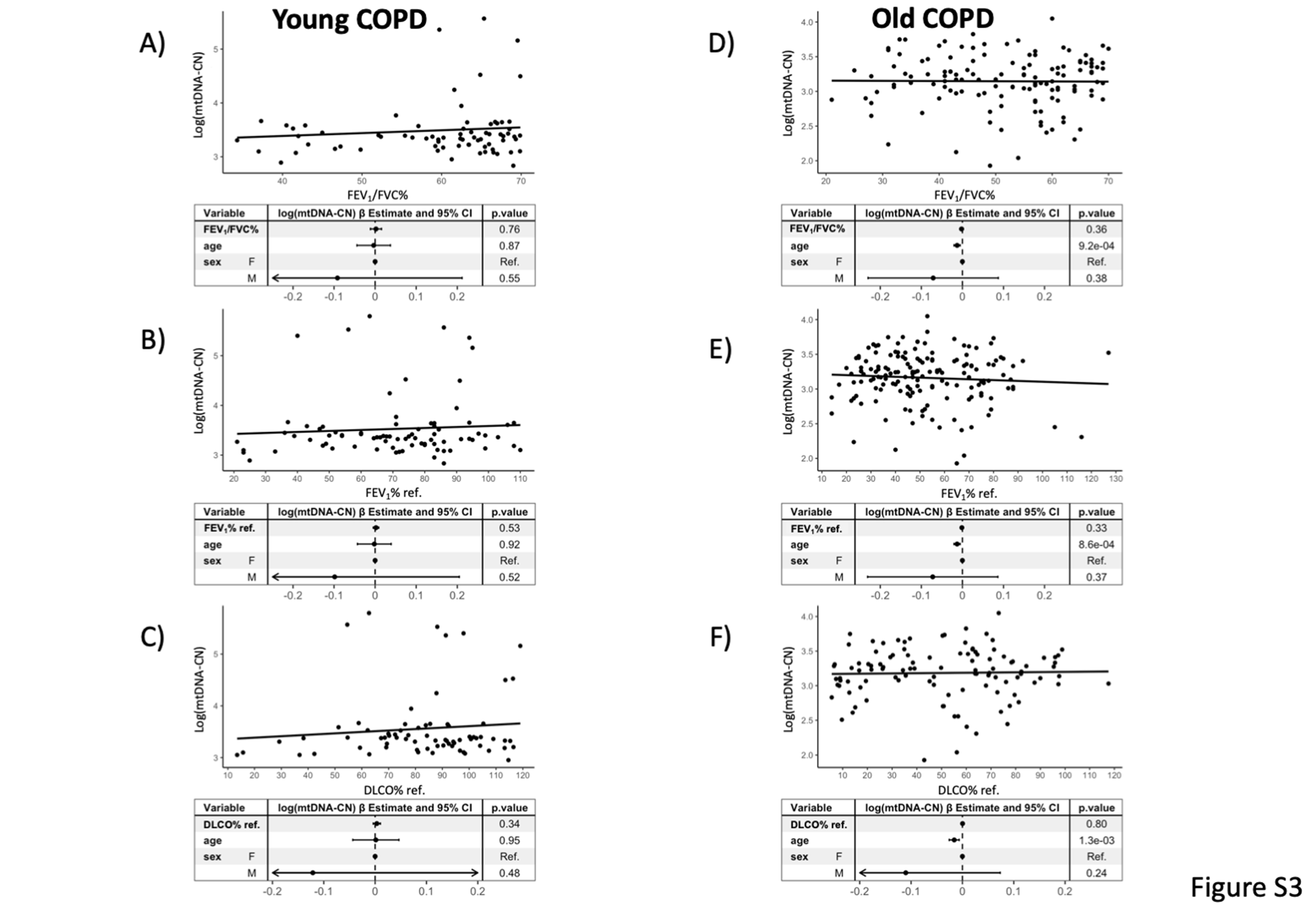

Supplement: Supplementary file 4 [file Image_3.TIFF]

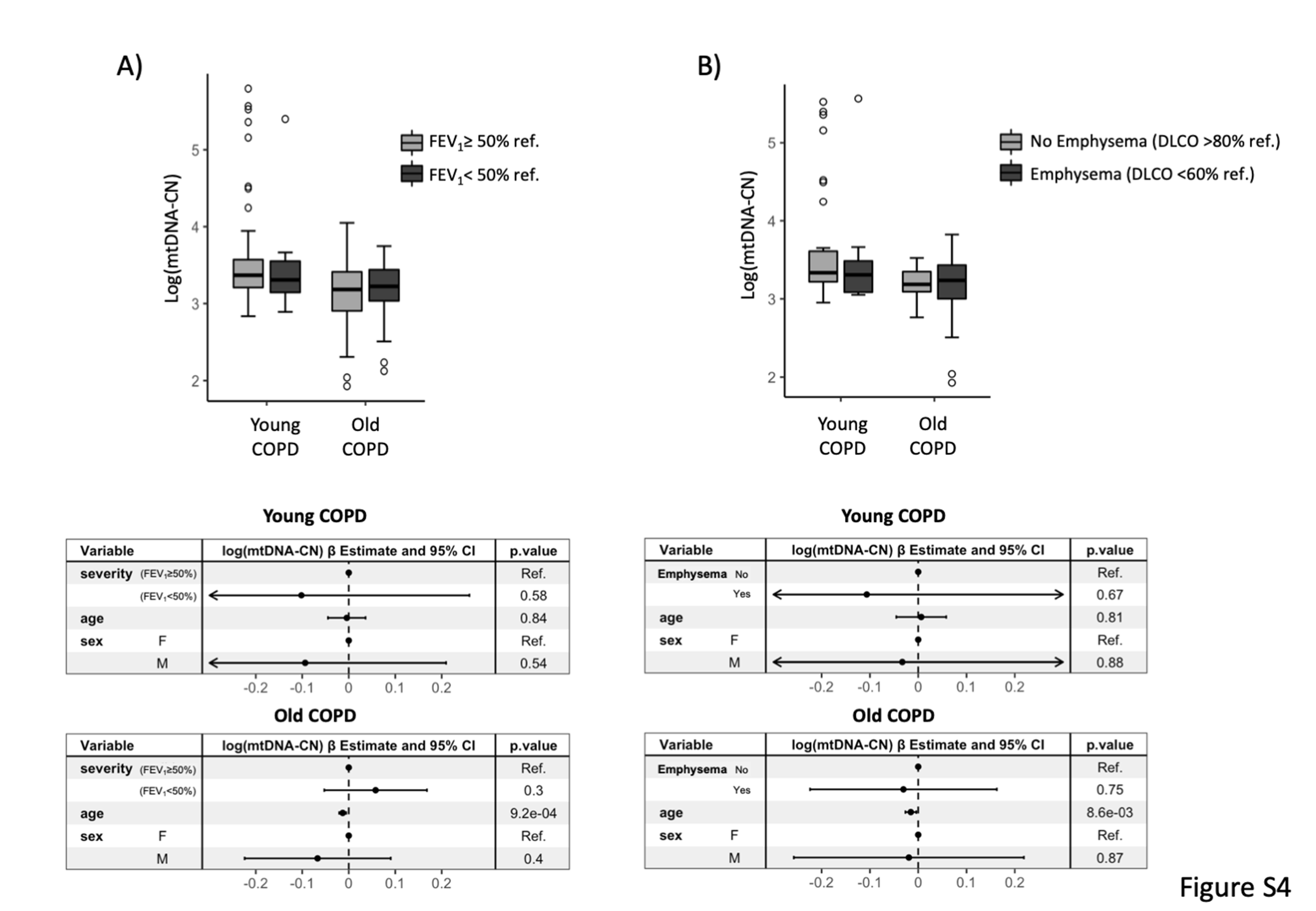

Supplement: Supplementary file 5 [file Image_4.TIFF]

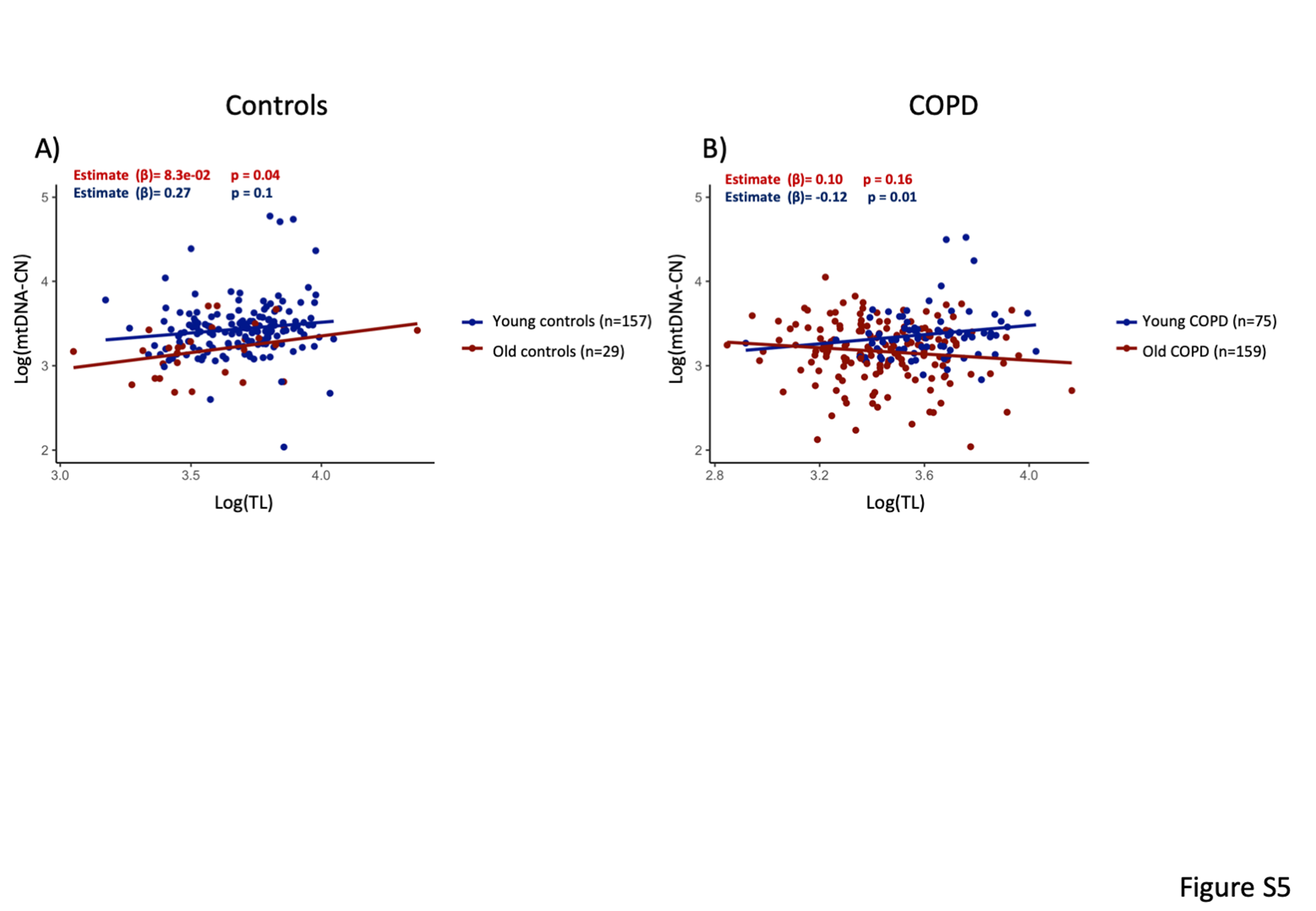

Supplement: Supplementary file 6 [file Image_5.TIFF]
